# SIRT1 deacetylates the cardiac transcription factor Nkx2.5 and inhibits its transcriptional activity

**DOI:** 10.1038/srep36576

**Published:** 2016-11-07

**Authors:** Xiaoqiang Tang, Han Ma, Lei Han, Wei Zheng, Yun-Biao Lu, Xiao-Feng Chen, Shu-Ting Liang, Gong-Hong Wei, Zhu-Qin Zhang, Hou-Zao Chen, De-Pei Liu

**Affiliations:** 1 State Key Laboratory of Medical Molecular Biology, Department of Biochemistry and Molecular Biology, Institute of Basic Medical Sciences, Chinese Academy of Medical Sciences and Peking Union Medical College, Beijing 100005, P.R. China; 2 Biocenter Oulu, Faculty of Biochemistry and Molecular Medicine, University of Oulu, Oulu, Finland

## Abstract

The homeodomain transcription factor Nkx2.5/Csx is critically essential for heart specification, morphogenesis, and homeostasis. Acetylation/deacetylation is important for the localization, stability and activation of transcription factors. It remains unknown how Nkx2.5 is deacetylated and how Nkx2.5 acetylation determines its activity. In this study, we provide evidence that the NAD^+^-dependent class III protein deacetylase SIRT1 deacetylates Nkx2.5 in cardiomyocytes and represses the transcriptional activity of Nkx2.5. We show that SIRT1 interacts with the C-terminus of Nkx2.5 and deacetylates Nkx2.5 at lysine 182 in the homeodomain. The mutation of Nkx2.5 at lysine 182 reduces its transcriptional activity. Furthermore, SIRT1 inhibits the transcriptional activity of Nkx2.5 and represses the expression of its target genes partly by reducing Nkx2.5 binding to its co-factors, including SRF and TBX5. Taken together, these findings demonstrate that SIRT1 deacetylates Nkx2.5 and inhibits the transcriptional activity of Nkx2.5.

In the cardiovascular system, diverse transcription factors conservatively regulate the functions of cardiomyocytes and their progenitor cells, and participate in cardiac development and homeostasis^1^. These transcription factors include Nkx2.5, GATAs, nuclear factor of activated T cells (NFATs), serum response factor (SRF), HAND, TBX and myocyte-specific enhancer-binding factors (MEFs)^2^. They control a cardiac gene program and therefore play a crucial role in transcription regulation during embryogenesis. In addition, cardiac transcription factors also regulate homeostasis and the development of heart diseases, such as congenital heart disease^3^.

The transcription factor Nkx2.5 is crucial for heart development and homeostasis, and mutations in this gene have been implicated in diverse congenital heart diseases and conduction defects in mouse models and humans^4^. Nkx2.5 directs the expression of target genes by interacting with its transcriptional co-factors^1^. A vast array of cardiac-specific ancillary proteins have been found to interact with Nkx2.5, including GATA4, HAND, TBX2, TBX5, TBX20, PITX2, and SRF^5^,^6^,^7^. For example, TBX5 associates with Nkx2.5 and synergistically promotes cardiomyocyte differentiation^8^. In addition, a physical association between Nkx2.5 and SRF activates cardiac-specific genes in cardiac cell lineages^9^.

Post-translational modifications (PTMs) also contribute to the localization, stability and transcriptional activity of Nkx2.5. Diverse PTMs of Nkx2.5 have been reported, including SUMOylation, O-linked N-acetylglucosamination and acetylation^5^,^10^,^11^. Wang *et al.* reported that SUMOylation at residue K51 of Nkx2.5 regulates Nkx2.5 DNA binding and its transcriptional activity^10^. In addition, high levels of O-GlcNAcylation causes downregulation of Nkx2.5^11^. A previous report showed that Nkx2.5 was acetylated by the protein acetyltransferase p300^5^; however, it remains unknown how Nkx2.5 is deacetylated. Our previous work demonstrated that the NAD^+^-dependent class III protein deacetylase SIRT1 is a target of Nkx2.5 and contributes to the protective function of Nkx2.5 in cardiomyocytes^12^. This finding prompted us to investigate whether SIRT1 can in turn deacetylate Nkx2.5.

In this study, we report that Nkx2.5 interacts with SIRT1 in cardiomyocytes. SIRT1 binds the C-terminus of Nkx2.5 and deacetylates it at lysine 182. SIRT1-mediated deacetylation of Nkx2.5 reduces its interaction with its co-factors (SRF and TBX5) and represses its transcriptional activity. Therefore, SIRT1 deacetylates Nkx2.5 and inhibits its transcriptional activity.

## Results

### SIRT1 physically interacts with Nkx2.5

**Figure 1:**
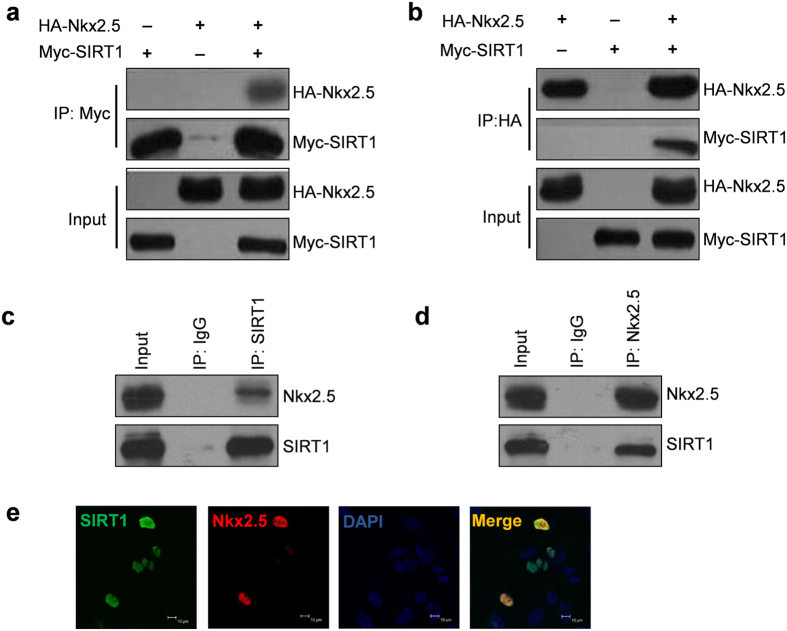
SIRT1 interacts with Nkx2.5. (**a,b**) Myc-SIRT1 interacts with HA-Nkx2.5 in HEK293A cells. Myc-SIRT1 and HA-Nkx2.5 were transfected individually or co-transfected into HEK293A cells for 48 hours. The cell lysates were immunoprecipitated with anti-Myc (**a**) or anti-HA (**b**) antibodies. Then, the immunoprecipitates were subjected to western blot analysis with the indicated antibodies. (**c,d**) SIRT1 interacts with Nkx2.5 in NRCMs. Primary NRCMs were cultured and lysed for immunoprecipitation with anti-SIRT1 (**c**) or anti-Nkx2.5 (**d**) antibodies or control IgG antibody. Then, western blot analysis was performed with the indicated antibodies. (**e**) Immunofluorescence assay showing SIRT1-Nkx2.5 co-localization in HEK293A cells. Myc-SIRT1 and HA-Nkx2.5 were co-transfected into HEK293A cells for 48 hours, and then immunofluorescence was performed to detect SIRT1 and Nkx2.5. Bar = 10 μm.

To determine whether SIRT1 can deacetylate Nkx2.5 and regulate its function, we first investigated the interaction between SIRT1 and Nkx2.5. We overexpressed HA-tagged Nkx2.5 (HA-Nkx2.5) and Myc-tagged SIRT1 (Myc-SIRT1) in HEK293A cells and performed an immunoprecipitation assay with anti-HA or anti-Myc antibodies to determine the interaction of SIRT1 and Nkx2.5. A strong interaction between SIRT1 and Nkx2.5 was detected (Fig. 1a,b). We further examined whether endogenous SIRT1 and Nkx2.5 could interact with each other. Therefore, we isolated and cultured neonatal rat cardiomyocytes (NRCMs) and performed an immunoprecipitation assay with anti-SIRT1 or anti-Nkx2.5 antibodies. We found that endogenous SIRT1 and Nkx2.5 interacted with each other in NRCMs (Fig. 1c,d). In addition, we performed an immunofluorescence assay to determine the co-localization of HA-Nkx2.5 and Myc-SIRT1 in HEK293A cells. The results showed a significant co-localization between Nkx2.5 and SIRT1 (Fig. 1e). Taken together, these findings demonstrate that SIRT1 can physically interact with Nkx2.5.

### SIRT1 directly interacts with Nkx2.5

**Figure 2:**
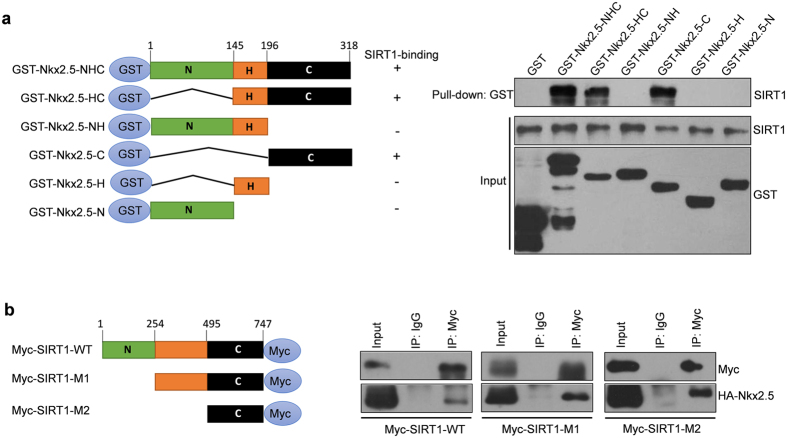
SIRT1 binds to the C-terminus of Nkx2.5. (**a**) Schematic presentation of GST-tagged wide-type (NHC) and deletion mutants of Nkx2.5 (left). Purified GST-tagged wide-type and mutated Nkx2.5 and MBP-tagged SIRT1 were subjected to a GST pull-down assay. Western blot assays were performed with the indicated antibodies (right). N: N-terminus; H: Homodomain; and C: C-terminus. (**b**) Schematic representation of Myc-tagged wide-type and deletion mutants of SIRT1 (M1 and M2, left). Wide type and mutated Myc-SIRT1 expressing plasmids were co-transfected with HA-Nkx2.5 into HEK293A cells for 48 hours. Immunoprecipitation and western blot assays were performed with the indicated antibodies (right). N: N-terminus; C: C-terminus. M1: Mutation 1; M2: Mutation 2.

We next performed a glutathione S-transferase (GST) pull-down assay to determine whether SIRT1 directly binds Nkx2.5 and which domain(s) of Nkx2.5 bind SIRT1. Purified GST-tagged wide-type Nkx2.5, various GST-tagged Nkx2.5 deletion mutants and purified maltose-binding protein-tagged SIRT1 were subjected to the GST pull-down assay. The results showed that Nkx2.5 directly bound SIRT1 and SIRT1 bound the C-terminus of Nkx2.5 (Fig. 2a), which is considered to have an inhibitory effect on the protein activity of Nkx2.5^13^. Interestingly, the immunoprecipitation assay showed that the C-terminus of SIRT1 was sufficient for Nkx2.5 binding (Fig. 2b).

### SIRT1 deacetylates Nkx2.5 *in vitro* and *in vivo*

**Figure 3:**
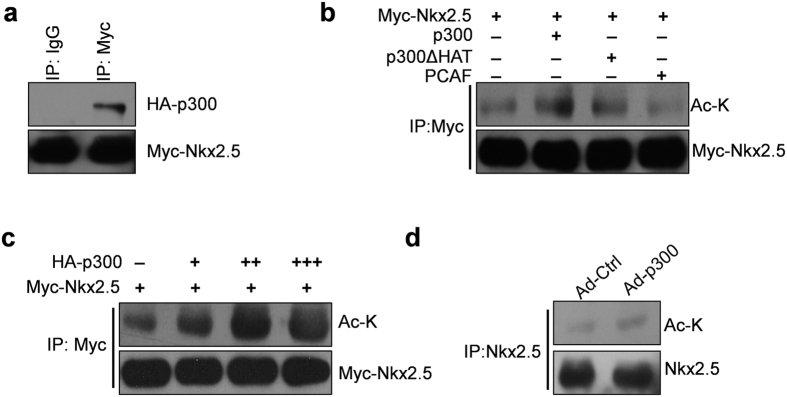
P300 interacts with and acetylates Nkx2.5. (**a**) HA-p300 and Myc-Nkx2.5 were co-transfected into HEK293A cells for 48 hours. The cell lysates were immunoprecipitated with anti-Myc or control IgG antibodies. The immunoprecipitates were then subjected to western blot analysis with the indicated antibodies. (**b**) Myc-Nkx2.5 was transfected into HEK293A cells without or with p300, p300ΔHAT or PCAF for 48 hours. The cell lysates were immunoprecipitated with anti-Myc antibody, followed by western blot with the indicated antibodies. (**c**) Myc-Nkx2.5 was transfected into HEK293A cells with or without 1 μg (+), 3 μg (++), or 5 μg (+++) of HA-p300 plasmid. The cell lysates were immunoprecipitated with anti-Myc antibody, followed by western blot with the indicated antibodies. (**d**) Primary neonatal rat cardiomyocytes were infected with adenovirus carrying p300 (Ad-p300) or control GFP (Ad-Ctrl) for 48 hours. The cell lysates were immunoprecipitated with an anti-Nkx2.5 antibody, followed by western blot with the indicated antibodies.

**Figure 4:**
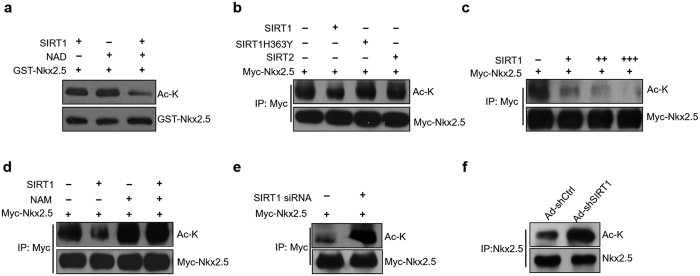
Nkx2.5 is a target of SIRT1-dependent deacetylation. (**a**) GST-Nkx2.5 and p300 were overexpressed in HEK293A cells, and then GST-Nkx2.5 was purified and subjected to an *in vitro* deacetylation assay in the presence/absence of recombinant SIRT1 and NAD. (**b**) Myc-Nkx2.5 was co-transfected with SIRT1, mutant SIRT1H363Y or SIRT2 into HEK293A cells for 48 hours, and then immunoprecipitation and western blot were performed with the indicated antibodies. (**c**) Myc-Nkx2.5 was co-transfected with increasing amounts of SIRT1 plasmid into HEK293A cell for 48 hours, and then immunoprecipitation and western blot were performed with the indicated antibodies. (**d**) Myc-Nkx2.5 was co-transfected with SIRT1 into HEK293A cells and the cells were then treated without or with 5 mM nicotinamide (NAM) for 48 hours. Immunoprecipitation and western blot were performed with the indicated antibodies. (**e**) Myc-Nkx2.5 was co-transfected without or with SIRT1 siRNA into HEK293A cells for 48 hours, and then immunoprecipitation and western blot were performed with the indicated antibodies. (**f**) Cardiomyocytes were infected with adenovirus-mediated shSIRT1 (Ad-shSIRT1) or control shRNA (Ad-shCtrl) for 48 hours, and then immunoprecipitation and western blot were performed with the indicated antibodies.

A previous work demonstrated that p300 acetylated Nkx2.5^5^. Consistently, we confirmed that Nkx2.5 interacts with p300 when overexpressed in HEK293A cells (Fig. 3a). We further showed that Nkx2.5 acetylation was increased by p300 but not by mutated p300 (p300∆HAT) or PCAF (another acetyltransferase) in HEK293A cells, and p300-mediated acetylation of Nkx2.5 was dose-dependent (Fig. 3b,c; Supplementary Fig. 1). PCAF is an acetyltransferase that acetylates diverse histone and non-histone substrates^14^. However, PCAF was not able to deacetylate Nkx2.5, which indicated that Nkx2.5 is a specific acetylation substrate of p300. In addition, we found that the acetylation of Nkx2.5 was increased by overexpressing p300 in NRCMs (Fig. 3d; Supplementary Fig. 2). Given that SIRT1 is a known deacetylase and is able to interact with Nkx2.5, we sought to determine whether SIRT1 could deacetylate Nkx2.5. We acetylated GST-Nkx2.5 by co-overexpressing p300 in HEK293A cells, purified GST-tagged Nkx2.5, and performed an *in vitro* deacetylation assay using recombinant human SIRT1. The results showed that Nkx2.5 was deacetylated by SIRT1 in the presence of NAD^+^ (Fig. 4a). Next, we tested the ability of SIRT1 to deacetylate Nkx2.5 in cells. Co-transfection of the SIRT1 and Nkx2.5 expression plasmids substantially decreased levels of acetylated Nkx2.5 (Fig. 4b). However, the enzymatic SIRT1 mutant (SIRT1H363Y) or SIRT2, which is another member of the Sirtuin family, did not deacetylate Nkx2.5 (Fig. 4b). In addition, the deacetylation of Nkx2.5 by SIRT1 was dose-dependent (Fig. 4c). Furthermore, we showed that either pharmacological inhibition of SIRT1 by nicotinamide (NAM) or siRNA-mediated SIRT1 knockdown increased the acetylation of Nkx2.5 in HEK293A cells (Fig. 4d,e). Finally, we investigated whether endogenous SIRT1 determined the acetylation of Nkx2.5 in NRCMs. We infected NRCMs with adenovirus that carrying SIRT1-targeting shRNA or control shRNA, and we found that SIRT1 knockdown increased the acetylation of endogenous Nkx2.5 in NRCMs (Fig. 4f). In conclusion, we demonstrate that SIRT1 is able to deacetylate Nkx2.5 in cardiomyocytes.

### SIRT1 deacetylates Nkx2.5 at lysine 182

**Figure 5:**
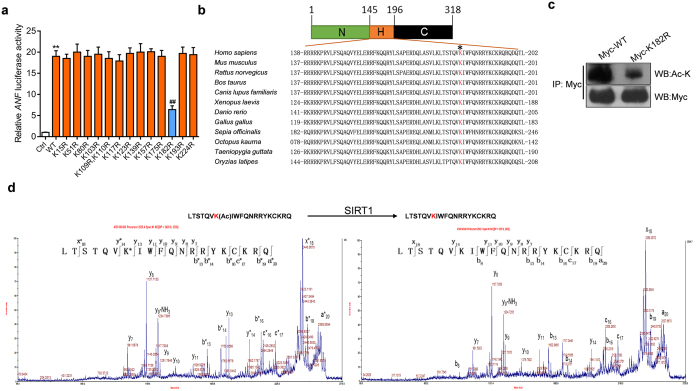
Lysine 182 of Nkx2.5 is deacetylated by SIRT1. (**a**) Luciferase assay showing that the mutation of lysine 182 reduces the transcriptional activity of Nkx2.5. pGL3-*ANF*-Luc plasmid was co-transfected with WT and mutant Nkx2.5 into HEK293A cells for 48 hours and then a luciferase assay was performed. **p < 0.01 *vs.* Ctrl, ^##^p < 0.01 *vs.* WT. The data are expressed as the means ± SEM of three independent experiments. (**b**) Multiple species contain a potentially conservatively acetylated lysine residue. The Nkx2.5 protein sequence from multiple species was BLASTed based on the reversibly acetylated lysine located at amino acid 182 (*) in mice. (**c**) Western blot showing that mutation of lysine 182 reduces the acetylation level of Nkx2.5. Myc-tagged wild type Nkx2.5 (Myc-WT) or mutant Nkx2.5-K182R (Myc-K182R) was expressed in HEK293A cells for 48 hours and were transfected into HEK293A cells for 48 hours and then immunoprecipitation and western blot were performed with the indicated antibodies. (**d**) Tandem mass spectrometry (MS/MS) analysis of Nkx2.5 peptide (AA residues 176–195) demonstrates that the acetylated lysine 182 of Nkx2.5 is deacetylated by SIRT1.

Next, we studied which lysine residues are deacetylated by SIRT1 and whether acetylation of Nkx2.5 influences its transcriptional activity. To this end, we mutated lysine (K) residues in Nkx2.5 to arginine (R) residues and screened the functional modification using a luciferase assay with the *ANF* promoter. Of the 13 Nkx2.5 mutations, only the K182R mutant had a significant decrease in transcriptional activity compared to the wide-type Nkx2.5 (Fig. 5a). Previous studies reported that mutations of Nkx2.5 at lysine 183 (lysine 182 in the mouse and rat Nkx2.5) are associated with congenital heart disease (CHD)^15^,^16^, which indicates that this site is critically important for normal cardiac function. In addition, this site is conserved across species (Fig. 5b). Therefore, we investigated whether this lysine site is acetylated. Indeed, the K182R (lysine (K) to arginine (R)) mutant resulted in a significant reduction in the acetylation of Nkx2.5 (Fig. 5c). To examine whether SIRT1 can deacetylate lysine 182, a fragment of the Nkx2.5 protein (residues 176–195) containing the acetylated lysine (lysine 182) was synthesized and incubated with purified SIRT1. Mass spectrometry (MS) analysis revealed that the acetyl group was removed by SIRT1 (Fig. 5d, see also the MS data in Supplementary Fig. 3 and the enlarged MS/MS data in Supplementary Fig. 4), indicating that lysine 182 of Nkx2.5 can be deacetylated by SIRT1. Altogether, these findings demonstrate that SIRT1 deacetylates Nkx2.5 at lysine 182, which determines the transcriptional activity of Nkx2.5.

### SIRT1 represses the expression of Nkx2.5 target genes

**Figure 6:**
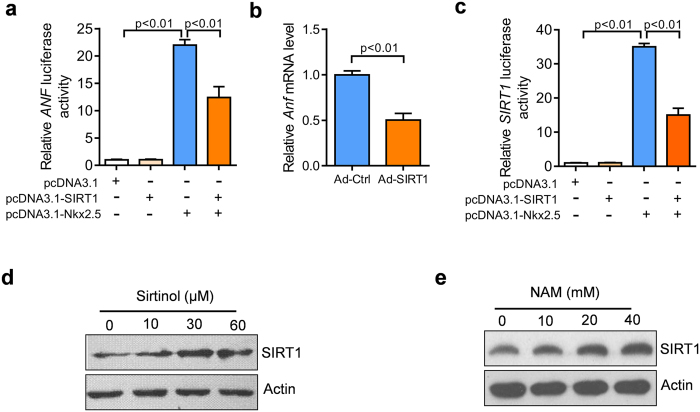
SIRT1 regulates the transcriptional activity of Nkx2.5. (**a**) SIRT1 overexpression represses Nkx2.5-mediated transactivation of the *ANF* promoter. pGL3-*ANF*-Luc and pRL-TK were co-transfected into HEK293A cells without or with Nkx2.5 and SIRT1 expression plasmids. Data are expressed as the means ± SEM of three independent experiments. (**b**) SIRT1 overexpression inhibits the expression of *Anf* in cardiomyocytes. NRCMs were infected with Ad-SIRT1 or Ad-Ctrl for 48 hours, and then the mRNA expression of *Anf* was analyzed using a qPCR assay. (**c**) SIRT1 overexpression represses Nkx2.5-mediated transactivation of the *SIRT1* promoter. pGL3-*SIRT1*-Luc and pRL-TK were co-transfected into HEK293A cells with Nkx2.5 and SIRT1 expression plasmid. The data are expressed as the mean ± SEM of three independent experiments. (**d**) Sirtinol increases the protein levels of SIRT1 in cardiomyocytes. NRCMs were treated with the indicated concentrations of Sirtinol for 48 hours, and then the protein levels of SIRT1 was analyzed using western blot. (**e**) Nicotinamide (NAM) increases the protein levels of SIRT1 in cardiomyocytes. NRCMs were treated with the indicated concentrations nicotinamide for 48 hours, and then the protein levels of SIRT1 were determined using western blot.

Given that SIRT1 deacetylates Nkx2.5 and the acetylation of Nkx2.5 determines its transcriptional activity, we wanted to know whether SIRT1 regulates the expression of Nkx2.5 target genes. Initially, we performed a luciferase assay to investigate whether SIRT1 regulates the effects of Nkx2.5 on the activity of the *ANF* promoter. The luciferase assay showed that Nkx2.5 promoted the activity of the *ANF* promoter, which was inhibited by SIRT1 overexpression (Fig. 6a). In rat cardiomyocytes, we found that adenovirus-mediated overexpression of SIRT1 repressed the mRNA expression of *Anf* (Fig. 6b). Our previous work demonstrated that *Sirt1* is a direct target for Nkx2.5. Nkx2.5 binds the promoter of *Sirt1* and activates its transcription in cardiomyocytes^12^. Therefore, we also determined the effect of SIRT1 protein on Nkx2.5-mediated activation of human *SIRT1* promoter. The results indicated that SIRT1 overexpression inhibited Nkx2.5-mediated promoter activation of human *SIRT1* (Fig. 6c). In addition, we inhibited the protein activity of endogenous SIRT1 protein with Sirtinol (a SIRT1 inhibitor) or NAM (a pan-Sirtuin inhibitor) in NRCMs, and found that SIRT1 inhibition promoted the expression of SIRT1 protein in NRCMs (Fig. 6d,e). These findings indicate that SIRT1 inhibits the transcriptional activity of Nkx2.5.

### SIRT1 represses the interaction between Nkx2.5 and its co-factors

**Figure 7:**
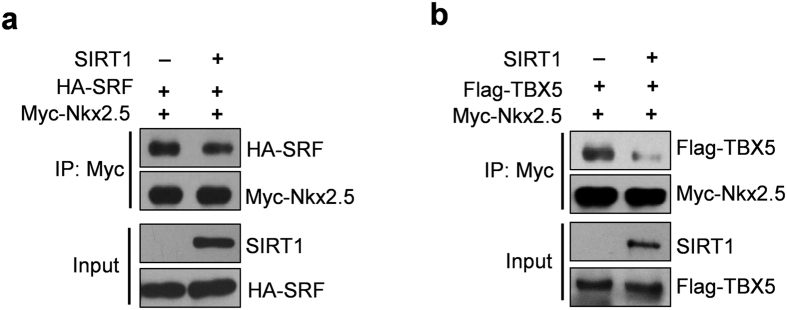
SIRT1 inhibits the interaction of Nkx2.5 with SRF and TBX5. (**a**) SIRT1 inhibits the interaction between Nkx2.5 and SRF. Nkx2.5 and SRF plasmids were co-transfected with/without SIRT1 into HEK293A cells for 48 hours, and then immunoprecipitation and western blot assays were performed with the indicated antibodies. (**b**) SIRT1 inhibits the interaction between Nkx2.5 and TBX5. Nkx2.5 and TBX5 plasmids were co-transfected with/without SIRT1 into HEK293A cells for 48 hours, and then immunoprecipitation and western blot assays were performed with the indicated antibodies.

Next, we explored how SIRT1 regulates the transcriptional activity of Nkx2.5. We performed an electrophoretic mobility shift assay (EMSA) to determine whether SIRT1 affects the DNA-binding capability of Nkx2.5. However, the results showed that SIRT1 or K182R mutation did not affect the DNA-binding capability of Nkx2.5 (Supplementary Fig. 5). Nkx2.5 interacts with its co-factors and promotes the transactivation of downstream genes^1^. We found that SIRT1 overexpression repressed the interaction between Nkx2.5 and its co-factors, including SRF and TBX5 (Fig. 7a,b; Supplementary Fig. 6). These results imply that SIRT1 regulates Nkx2.5 transcriptional activity, partly by reducing interactions with its co-factors.

## Discussion

Of the cardiac transcription factors, only GATA4 and Nkx2.5 were reported to be acetylated. GATA4 is acetylated by p300 and deacetylated by HDAC2^17^,^18^. p300 interacts with and acetylates GATA4. Both the acetylated form of GATA4 and the relative level of the p300/GATA4 complex are markedly increased during cardiac hypertrophy^17^,^19^. The specific reduction of p300 content or activity diminishes stress-induced hypertrophy and prevents the development of heart failure^19^. The chromatin-modifying enzyme HDAC2 functions with a small homeodomain factor, Hopx, to mediate deacetylation of GATA4, and these three factors coordinate to regulate cardiac myocyte proliferation during embryonic development^18^. GATA4 interacts with and targets SIRT1 to the *myogenin* promoter, which affects skeletal myogenic differentiation^20^. However, SIRT1 was not shown to modify GATA4 directly.

We found that SIRT1 deacetylates Nkx2.5 in cardiomyocytes. The evolutionally conserved homeobox transcription factor Nkx2.5/Csx has been in the forefront in the field of cardiac biology, providing molecular insights into the mechanisms of cardiac development and diseases^13^. p300 acts as a Nkx2.5 cofactor and increases Nkx2.5 activity by relieving the conformational impediment of its inhibitory C-terminal domain^5^. However, the deacetylation mechanism of Nkx2.5 remains unknown. Here we provide evidence that SIRT1 and p300 modulate Nkx2.5 acetylation in cardiomyocytes. We found that p300 but not PCAF acetylated Nkx2.5, which was deacetylated by SIRT1, but not SIRT2. Furthermore, we found that SIRT1 bound the C-terminus of Nkx2.5 and repressed its transcriptional activity. Therefore, Nkx2.5 is the first identified cardiac transcription factor to be regulated by SIRT1 in cardiomyocytes.

In humans, mutations of Nkx2.5 at lysine 183 (lysine 182 in the mouse and rat Nkx2.5) are associated with congenital heart disease^15^,^16^. Mutations at this site decreased the transcriptional activity of Nkx2.5. Our mass spectrometry analysis showed that the acetyl group of Nkx2.5 at lysine 182 was removed by SIRT1, implying that lysine 182 of Nkx2.5 can be deacetylated by SIRT1. Generally, enzymes modify their substrates at the domains where they bind. Nevertheless, distant modification is also common. For example, SIRT1 binds the N-terminus of hMOF and TIP60, but mediates deacetylation of the enzymatic domains of hMOF and TIP60 at C-terminus^21^,^22^. In addition, SIRT1 binds the C-terminus of p300. Interestingly, SIRT1 deacetylates p300 at its N-terminus^23^. Likewise, we found that SIRT1 binds Nkx2.5 at its C-terminus but modifies residue 182 of the N-terminal homeodomain. This finding also indicates that the C-terminus of Nkx2.5 is also important for regulating the N-terminus activity. Indeed, we also found that lysine 182 was important for the total acetylation level of Nkx2.5 and its transcriptional activity. In addition, Nkx2.5 was still significantly acetylated even when K182R was used, which indicates the presence of other acetylation sites. However, mutations at the other lysines of Nkx2.5 did not affect the transcriptional activity of Nkx2.5, indicating that acetylation of Nkx2.5 at K182 plays the major role in determining the transcriptional activity of Nkx2.5. Nevertheless, we still cannot exclude the possibility that acetylation of the other lysines may regulate the protein stability, translocation and other functions of Nkx2.5. All together, these findings demonstrate that SIRT1 binds the C-terminus of Nkx2.5 and deacetylates it at lysine 182, which determines the transcriptional activity of Nkx2.5. These findings provide new insights into the molecular mechanisms underlying Nkx2.5 mutations in congestive heart diseases.

The function of Nkx2.5 in cardiomyocytes largely depends on co-operation with its co-factors^1^. Our evidences showed that SIRT1 regulated the interaction between Nkx2.5 and its co-factors SRF and TBX5, but did not affect the DNA-binding ability of Nkx2.5. Other mechanisms may also contribute to the effect of SIRT1 on Nkx2.5. For example, acetylation and SUMOylation of proteins may link regulatory functions^24^,^25^. SIRT1-mediated deacetylation of Nkx2.5 may affect the SUMOylation of Nkx2.5. Wang *et al.* reported that SUMOylation at residue K51 of Nkx2.5 regulated Nkx2.5 DNA binding and its transcriptional activity^10^. However, they also showed that expression of the Nkx2.5 mutant (K51R) transgene in wild-type murine hearts did not result in any overt cardiac phenotype^26^. In addition, the work by Costa *et al.* showed in a range of cultured cell lines that the point mutation K51R did not affect Nkx2.5 activity or DNA binding^27^. Therefore, the function of SUMOylation at the K51 residue is under debate. In our work, we also mutated Nkx2.5 at K51 (K51R). Interestingly, we did not find any effects of this mutation on the transcriptional activity of *ANF* using a luciferase assay, which is consistent with the previous study by Costa *et al.*^27^. Therefore, SIRT1-mediated repression of Nkx2.5 transcriptional activity may not rely on the SUMOylation of K51, but partly through inhibition of the interaction between Nkx2.5 and its co-factors.

SIRT1 is expressed at high levels in prenatal hearts and is essential for cardiac development and prenatal survival^28^,^29^. In cardiomyocytes, SIRT1 overexpression inhibited the expression of Nkx2.5 target genes, in part through repressing the interaction between Nkx2.5 and its transcriptional co-factors. Our previous work demonstrated that Nkx2.5 could bind the promoter of *Sirt1* and induce its transcription. Nkx2.5 promotes the expression of *Sirt1*, which contributes to Nkx2.5-mediated cardiomyocyte survival^12^. Interestingly, SIRT1 in turn deacetylates Nkx2.5 and represses the transcriptional activity of Nkx2.5. Therefore, the Nkx2.5/SIRT1 negative feedback regulatory loop may potentially be involved in cardiomyocyte survival during cardiogenesis and congenital heart diseases.

In summary, we provide evidence that SIRT1 deacetylates the cardiac transcription factor Nkx2.5 and represses its transcriptional activity. SIRT1 binds the C-terminus of Nkx2.5 and deacetylates Nkx2.5 at lysine 182. SIRT1-mediated deacetylation of Nkx2.5 controls its transcriptional activity in part through inhibiting the binding of Nkx2.5 to its co-factors.

## Methods

### Cell lines and culture

HEK293A cells were cultured in Dulbecco’s modified Eagle’s medium (DMEM; Invitrogen) supplemented with 10% fetal bovine serum (FBS; Invitrogen) and 1% penicillin-streptomycin mixture. Primary neonatal rat cardiomyocytes (NRCMs) were prepared from Sprague-Dawley rats of 1–3 day-old. All animal protocols were approved by the Animal Care and Use Committee at the Institute of Basic Medical Sciences, Chinese Academy of Medical Sciences and Peking Union Medical College (CAMS & PUMC). The methods were carried out in accordance with the relevant guidelines. In detail, PBS containing 0.03% trypsin and 0.04% collagenase type II was used to isolate cardiomyocytes, followed by fibroblast removal using a differential attachment technique. The NRCMs were seeded at a density of 2 × 10^5^ cells per well onto six-well culture plates coated with gelatin in plating medium, which consisted of DMEM supplemented with 10% fetal calf serum, 0.1 mM 5-bromodeoxyuridine (to inhibit fibroblast proliferation) and penicillin/streptomycin^30^. Then, the cells were cultured in serum-free DMEM for additional 24 hours before a certain experiment was performed. The NRCMs were treated with nicotinamide (Sigma) or Sirtinol (Sigma) as indicated in the figure legends. All the cells were cultured at 37 °C with 5% CO_2_. Transfection of HEK293A cells was performed with VigoFect transfection kit (Vigorous Biotechnology) according to the manufacturer protocol.

### Plasmids and adenovirus

The pTA-Luc plasmid containing a fragment (-2852 bp - +1 bp) of the SIRT1 promoter was kindly provided by Dr. Toren Finkel. The *SIRT1* promoter fragment was amplified by PCR and inserted into a pGL3-basic plasmid (Promega). The *ANF*-Luc plasmid was kindly provided by Dr. Eric N. Olson. Full-length cDNAs including mouse *Nkx2.5* (a gift from Dr. Eric N. Olson), and human *SIRT1* (a gift from Dr. Ishikawa) were sequenced and subcloned into a pcDNA3.1 expression vector. *Nkx2.5* deletion mutant expression vectors were constructed by inserting the mouse *Nkx2.5* cDNA into the pcDNA3.1 vector. Site-directed mutagenesis was carried out using the QuikChange Site-Directed Mutagenesis Kit (Stratagene). The expression construct pcDNA3.1 contains Myc, and a Flag or HA tag.

Replication-defective adenoviral vectors expressing SIRT1 (Ad-SIRT1), p300 (Ad-p300) or control green fluorescent protein (Ad-Ctrl) in addition to a vector for adenovirus-mediated knockdown of SIRT1 (Ad-shSIRT1) or a control shRNA vector (Ad-shCtrl) were generated using the AdEasy Vector kit (Quantum Biotechnologies) according to previously described methods^31^.

### Immunoprecipitation (IP) and western blot

Immunoprecipitation analysis was performed using standard protocols based on previously described methods^22^. Cultured cells were lysed with Cell Lysis Buffer (Beyotime) supplemented with a protease inhibitor cocktail (Roche). A total of 40 μg protein were separated on a 12% SDS–polyacrylamide gel (SDS-PAGE). After electrophoresis, the proteins were transferred to PVDF membranes, followed by antigen-blocking in 5% fat-free milk. The membranes were then probed with the indicated antibodies overnight at 4 °C, and then washed and incubated with primary-antibody-matched and HRP-conjugated secondary antibodies (Zhongsanjinqiao) for 2 hours. Finally, the membranes were washed and visualized using Chemiluminescent ECL reagent (Vigorous Biotechnology). The following primary antibodies were used: anti-Myc antibody (Santa Cruz Biotechnology), anti-GST antibody (Santa Cruz Biotechnology), anti-SIRT1 antibody (Santa Cruz Biotechnology), anti-Nkx2.5 antibody (Santa Cruz Biotechnology), anti-HA antibody (Santa Cruz Biotechnology), anti-Flag antibody (Sigma), anti-acetylated lysine (ac-K) antibody (Cell Signaling Technology), and anti-actin antibody (Santa Cruz Biotechnology).

### *In vitro* deacetylation assay

The *in vitro* deacetylation assay was performed using the standard protocols based on previously described methods^30^. Briefly, acetylated GST-Nkx2.5 (purified from HEK293A cells) was incubated in deacetylation buffer (25 mM Tris-HCl, pH 8.0,137 mM NaCl, 2.7 mM KCl and 1 mM MgCl_2_) in the presence of purified recombinant human SIRT1 (3.5 U; Sigma) and in the presence or absence of NAD^+^ (60 μM; Sigma) at 30 °C for 1 hour. The products of the reaction were resolved using SDS-PAGE and analyzed using western blot.

### Quantitative real-time polymerase chain reaction (q-PCR)

Total RNA was extracted from cultured cells with TRIzol (Invitrogen) from cultured cells. A total of 1 μg of total RNA was subjected to cDNA synthesis with a ProtoScript^®^ First Strand cDNA Synthesis Kit (New England BioLabs). The cDNA was then subjected to qPCR assay using the SYBR Green Master Mix Kit (TaKaRa). The primers used in the PCR reactions were as follows:

rat *Anf* forward primer, 5′-GAAGATGCCGGTAGAAGATGAG-3′;

rat *Anf* reverse primer, 5′-AGAGCCCTCAGTTTGCTTTTC-3′;

rat *Actin* forward primer, 5′-CGTGAAAAGATGACCCAGAT-3′;

rat *Actin* reverse primer, 5′-ATTGCCGATAGTGATGACCT-3′.

### Immunofluorescence assay

The immunofluorescence assay was performed according to previously methods^32^. Briefly, HEK293A cells expressing Myc-SIRT1 and HA-Nkx2.5 were fixed in 4% paraformaldehyde at room temperature for 10 min. Post-fixative permeabilization was carried out in PBS containing 0.1% Triton X-100 for 10 min. The expression of Myc-SIRT1 or HA-Nkx2.5 was detected using mouse anti-Myc and rabbit anti-HA antibodies, followed by anti-rabbit IgG/TRITC (Invitrogen) and anti-mouse IgG/FITC (Invitrogen) staining. The nuclei were stained with DAPI (Sigma). The images were captured with an Olympus FV1000MPE and analyzed using F1000 Viewer software.

### Pull-down assay

GST-Nkx2.5 or GST-Nkx2.5 deletion mutants and maltose-binding protein-tagged (MBP) SIRT1 were expressed and purified from bacteria using standard protocols based on previously described methods^22^. The coding sequence of Nkx2.5 or SIRT1 was ligated into pGEX-KG in fusion with GST or MBP tag for prokaryotic expression and into pMSCV-puro in fusion with GST tag for eukaryotic expression using previously described methods^22^. Equimolar quantities of purified GST-labeled proteins were conjugated to glutathione-Sepharose beads and incubated with purified MBP labeled proteins at 4 °C for 16 hours. After five washes, the proteins were eluted and resolved using western blot with anti-SIRT1 or anti-Nkx2.5 antibodies.

### Luciferase assay

HEK293A cells were cultured in triplicate to 80% confluency in 24-well plates and co-transfected with the reporter constructs (pGL3-*ANF*-Luc or pGL3-*SIRT1*-Luc), the expression vectors (SIRT1 or control expressing vectors) and the pRL-TK-Luc internal control plasmid as indicated. Luciferase activity was assessed using a Dual-luciferase Reporter Assay System (Promega).

### Electrophoretic mobility shift assay (EMSA)

Double-stranded probes were generated by annealing equimolar complementary oligonucleotides in 50 mM Tris-HCl (pH 7.4), 1 mM EDTA, 100 mM NaCl, and 13 mM MgCl_2_ as follows: 88 °C for 2 min, 65 °C for 10 min, 37 °C for 10 min, and 25 °C for 5 min. The double-stranded oligonucleotides were end-labeled with [r-32P] ATP (3000 mCi/mmol) and T4 polynucleotide kinase. Binding assays contained 32P-labeled oligonucleotide (0.3 ng), 5 μg of nuclear proteins and 1 μg of herring sperm DNA (Promega), and they were adjusted to 20 μl with binding buffer (20 mM HEPES, pH 7.9, 1.5 mM MgCl_2_, 0.2 mM EDTA, 1 mM dithiothreitol, 0.1 mM phenylmethylsulfonyl fluoride and 7.5% glycerol). Binding reactions were carried out at room temperature for 30 min. An aliquot (15 μl) of each reaction was loaded onto a 5% nondenaturing polyacrylamide gel, which was electrophoresed in 0.5X TBE buffer at 120 V. Following electrophoresis, the gels were dried and autoradiographed. In the competition assays, unlabeled competitor oligonucleotides were added at 100-fold excess before the addition of the 32P-labeled probe. For super-shift assay, anti-Nkx2.5 antibody was used. The oligonucleotide probes used in the EMSAs are as follows:

*ANF* antisense, 5′-TGATTTGCCTCAAGAGGCCCCCACTTCAAAGGTGTGA-3′ and

*ANF* sense, 5′-TCACACCTTTGAAGTGGGGGCCTCTTGAGGCAAATCA-3′.

### Statistical analysis

The values were expressed as the means ± SEM of at least three independent experiments if no additional information was indicated. All the experiments have been repeated at least for three times. Student’s *t* test was applied to analyze difference between two groups and one-way ANOVA followed by Bonferroni *post-hoc* test was used to analyze the difference among multiple groups. The statistical analysis was performed with GraphPad Prism 6. *P* values of less than 0.05 were considered to be statistically significant.

## Additional Information

**How to cite this article**: Tang, X. *et al.* SIRT1 deacetylates the cardiac transcription factor Nkx2.5 and inhibits its transcriptional activity. *Sci. Rep.* **6**, 36576; doi: 10.1038/srep36576 (2016).

**Publisher’s note:** Springer Nature remains neutral with regard to jurisdictional claims in published maps and institutional affiliations.

## Supplementary Material

Supplementary Information

## References

[b1] Akazawa H. & Komuro I. Roles of Cardiac Transcription Factors in Cardiac Hypertrophy. Circ. Res. 92, 1079–1088 (2003). 12775656 10.1161/01.RES.0000072977.86706.23

[b2] Mudd J. O. & Kass D. A. Tackling heart failure in the twenty-first century. Nature 451, 919–928 (2008). 18288181 10.1038/nature06798

[b3] McCulley D. J. & Black B. L. Transcription factor pathways and congenital heart disease. Curr. Top. Dev. Biol. 100, 253 (2012). 22449847 10.1016/B978-0-12-387786-4.00008-7 PMC3684448

[b4] Costa M. W. *et al.* Functional Characterization of a Novel Mutation in NKX2-5 Associated With Congenital Heart Disease and Adult-Onset Cardiomyopathy. Circ. Cardiovasc. Genet. 6, 238–247 (2013). 23661673 10.1161/CIRCGENETICS.113.000057 PMC3816146

[b5] Li T. *et al.* Carboxyl Terminus of NKX2.5 Impairs its Interaction with p300. J. Mol. Biol. 370, 976–992 (2007). 17544441 10.1016/j.jmb.2007.05.033

[b6] Moskowitz I. P. *et al.* A molecular pathway including Id2, Tbx5, and Nkx2-5 required for cardiac conduction system development. Cell 129, 1365–1376 (2007). 17604724 10.1016/j.cell.2007.04.036

[b7] Schlesinger J. *et al.* The cardiac transcription network modulated by Gata4, Mef2a, Nkx2. 5, Srf, histone modifications, and microRNAs. PLoS Genet. 7, e1001313 (2011). 21379568 10.1371/journal.pgen.1001313 PMC3040678

[b8] Hiroi Y. *et al.* Tbx5 associates with Nkx2-5 and synergistically promotes cardiomyocyte differentiation. Nat. Genet. 28, 276–280 (2001). 11431700 10.1038/90123

[b9] Chen C. Y. & Schwartz R. J. Recruitment of the tinman homolog Nkx-2.5 by serum response factor activates cardiac alpha-actin gene transcription. Mol. Cell. Biol. 16, 6372–6384 (1996). 8887666 10.1128/mcb.16.11.6372 PMC231639

[b10] Wang J., Zhang H., Iyer D., Feng X.-H. & Schwartz R. J. Regulation of Cardiac Specific nkx2.5 Gene Activity by Small Ubiquitin-like Modifier. J. Biol. Chem. 283, 23235–23243 (2008). 18579533 10.1074/jbc.M709748200 PMC2516993

[b11] Kim H. S., Woo J. S., Joo H. J. & Moon W. K. Cardiac transcription factor Nkx2.5 is downregulated under excessive O-GlcNAcylation condition. PLoS One 7, e38053 (2012). 22719862 10.1371/journal.pone.0038053 PMC3376112

[b12] Zheng W. *et al.* SIRT1 mediates the protective function of Nkx2.5 during stress in cardiomyocytes. Basic Res. Cardiol. 108, 364 (2013). 23744058 10.1007/s00395-013-0364-y

[b13] Akazawa H. & Komuro I. Cardiac transcription factor Csx/Nkx2-5: Its role in cardiac development and diseases. Pharmacol. Ther. 107, 252–268 (2005). 15925411 10.1016/j.pharmthera.2005.03.005

[b14] Menzies K. J., Zhang H., Katsyuba E. & Auwerx J. Protein acetylation in metabolism-metabolites and cofactors. Nat. Rev. Endocrinol. 12, 43–60 (2016). 26503676 10.1038/nrendo.2015.181

[b15] Reamon-Buettner S. M. & Borlak J. Somatic NKX2-5 mutations as a novel mechanism of disease in complex congenital heart disease. J. Med. Genet. 41, 684–690 (2004). 15342699 10.1136/jmg.2003.017483 PMC1735891

[b16] Reamon-Buettner S. M. *et al.* Novel NKX2–5 Mutations in Diseased Heart Tissues of Patients with Cardiac Malformations. Am. J. Pathol. 164, 2117–2125 (2004). 15161646 10.1016/S0002-9440(10)63770-4 PMC1615780

[b17] Wei J. Q. *et al.* Quantitative control of adaptive cardiac hypertrophy by acetyltransferase p300. Circulation 118, 934–946 (2008). 18697823 10.1161/CIRCULATIONAHA.107.760488 PMC2726266

[b18] Trivedi C. M. *et al.* Hopx and Hdac2 interact to modulate Gata4 acetylation and embryonic cardiac myocyte proliferation. Dev. Cell 19, 450–459 (2010). 20833366 10.1016/j.devcel.2010.08.012 PMC2947937

[b19] Morimoto T. *et al.* The dietary compound curcumin inhibits p300 histone acetyltransferase activity and prevents heart failure in rats. J. Clin. Invest. 118, 868–878 (2008). 18292809 10.1172/JCI33160 PMC2248328

[b20] Wang L. *et al.* GATA-binding protein 4 (GATA-4) and T-cell acute leukemia 1 (TAL1) regulate myogenic differentiation and erythropoietin response via cross-talk with Sirtuin1 (Sirt1). J. Biol. Chem. 287, 30157–30169 (2012). 22773876 10.1074/jbc.M112.376640 PMC3436270

[b21] Peng L. *et al.* SIRT1 Negatively Regulates the Activities, Functions, and Protein Levels of hMOF and TIP60. Mol. Cell. Biol. 32, 2823–2836 (2012). 22586264 10.1128/MCB.00496-12 PMC3416197

[b22] Lu L. *et al.* Modulations of hMOF autoacetylation by SIRT1 regulate hMOF recruitment and activities on the chromatin. Cell Res. 21, 1182–1195 (2011). 21502975 10.1038/cr.2011.71 PMC3193486

[b23] Bouras T. *et al.* SIRT1 Deacetylation and Repression of p300 Involves Lysine Residues 1020/1024 within the Cell Cycle Regulatory Domain 1. J. Biol. Chem. 280, 10264–10276 (2005). 15632193 10.1074/jbc.M408748200

[b24] Yang Y. *et al.* Acetylated hsp70 and KAP1-mediated Vps34 SUMOylation is required for autophagosome creation in autophagy. Proc. Natl. Acad. Sci. USA 110, 6841–6846 (2013). 23569248 10.1073/pnas.1217692110 PMC3637746

[b25] Yang S. H. & Sharrocks A. D. Ubc9 acetylation: a new route for achieving specificity in substrate SUMOylation. EMBO J. 32, 773–774 (2013). 23395903 10.1038/emboj.2013.21 PMC3604717

[b26] Kim E. Y. *et al.* Expression of Sumoylation Deficient Nkx2.5 Mutant in Nkx2.5 Haploinsufficient Mice Leads to Congenital Heart Defects. PLoS One 6, e20803 (2011). 21677783 10.1371/journal.pone.0020803 PMC3108998

[b27] Costa M. W. *et al.* Complex SUMO-1 regulation of cardiac transcription factor Nkx2-5. PLoS One 6, e24812 (2011). 21931855 10.1371/journal.pone.0024812 PMC3171482

[b28] Cheng H. L. *et al.* Developmental defects and p53 hyperacetylation in Sir2 homolog (SIRT1)-deficient mice. Proc. Natl. Acad. Sci. USA 100, 10794–10799 (2003). 12960381 10.1073/pnas.1934713100 PMC196882

[b29] Mu W. *et al.* Overexpression of a dominant-negative mutant of SIRT1 in mouse heart causes cardiomyocyte apoptosis and early-onset heart failure. Sci. China Life Sci. 57, 915–924 (2014). 25104317 10.1007/s11427-014-4687-1

[b30] Luo Y.-X. *et al.* Sirt4 accelerates Ang II-induced pathological cardiac hypertrophy by inhibiting manganese superoxide dismutase activity. Eur. Heart J. (2016). 10.1093/eurheartj/ehw138 27099261

[b31] Zhang Z. Q. *et al.* Epigenetic regulation of NKG2D ligands is involved in exacerbated atherosclerosis development in Sirt6 heterozygous mice. Sci. Rep. 6, 23912 (2016). 27045575 10.1038/srep23912 PMC4820703

[b32] Pei J.-F. *et al.* Human paraoxonase gene cluster overexpression alleviates angiotensin II-induced cardiac hypertrophy in mice. Sci. China Life Sci. (2016). 10.1007/s11427-016-0131-4 27578362

