# Supplementary material for: SIRT1 deacetylates the cardiac transcription factor Nkx2.5 and inhibits its transcriptional activity
Source: Sci Rep. 2016 Nov 7;6:36576. doi: 10.1038/srep36576 (PMC5098195; doi:10.1038/srep36576)
Supplement: Supplementary Information [file srep36576-s1.pdf]

Supplementary information

SIRT1 deacetylates the cardiac transcription factor Nkx2.5 and inhibits its transcriptional activity

Xiaoqiang Tang[1,*], Han Ma[1,*], Lei Han[1,*], Wei Zheng[1], Yun-Biao Lu[1], Xiao-Feng Chen[1], Shu-Ting Liang[1], Gong-Hong Wei[2], Zhu-Qin Zhang[1], Hou-Zao Chen[1], De-Pei Liu[1]

[1] State Key Laboratory of Medical Molecular Biology, Department of Biochemistry and Molecular Biology, Institute of Basic Medical Sciences, Chinese Academy of Medical Sciences and Peking Union Medical College, Beijing 100005, P.R. China.

[2] Faculty of Biochemistry and Molecular Medicine, University of Oulu, Oulu, Finland.

* These authors contributed equally to this work.

Correspondence and requests for materials should be addressed to H.-Z.C. (email: chenhouzao@ibms.cams.cn) or D.-P.L. (email: liudp@pumc.edu.cn)

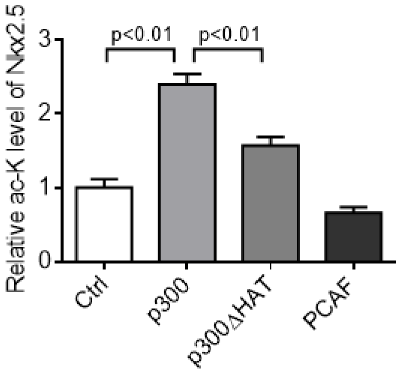

Supplementary Figure 1 Relative acetylated lysine (ac-K) level of Nkx2.5 (related to Figure 3b).

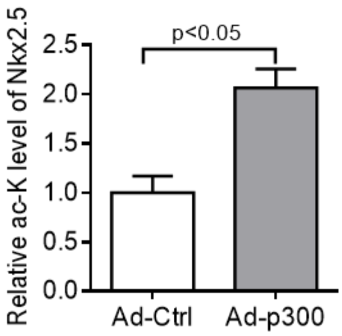

Supplementary Figure 2 Relative acetylated lysine (ac-K) level of Nkx2.5 (related to Figure 3d).

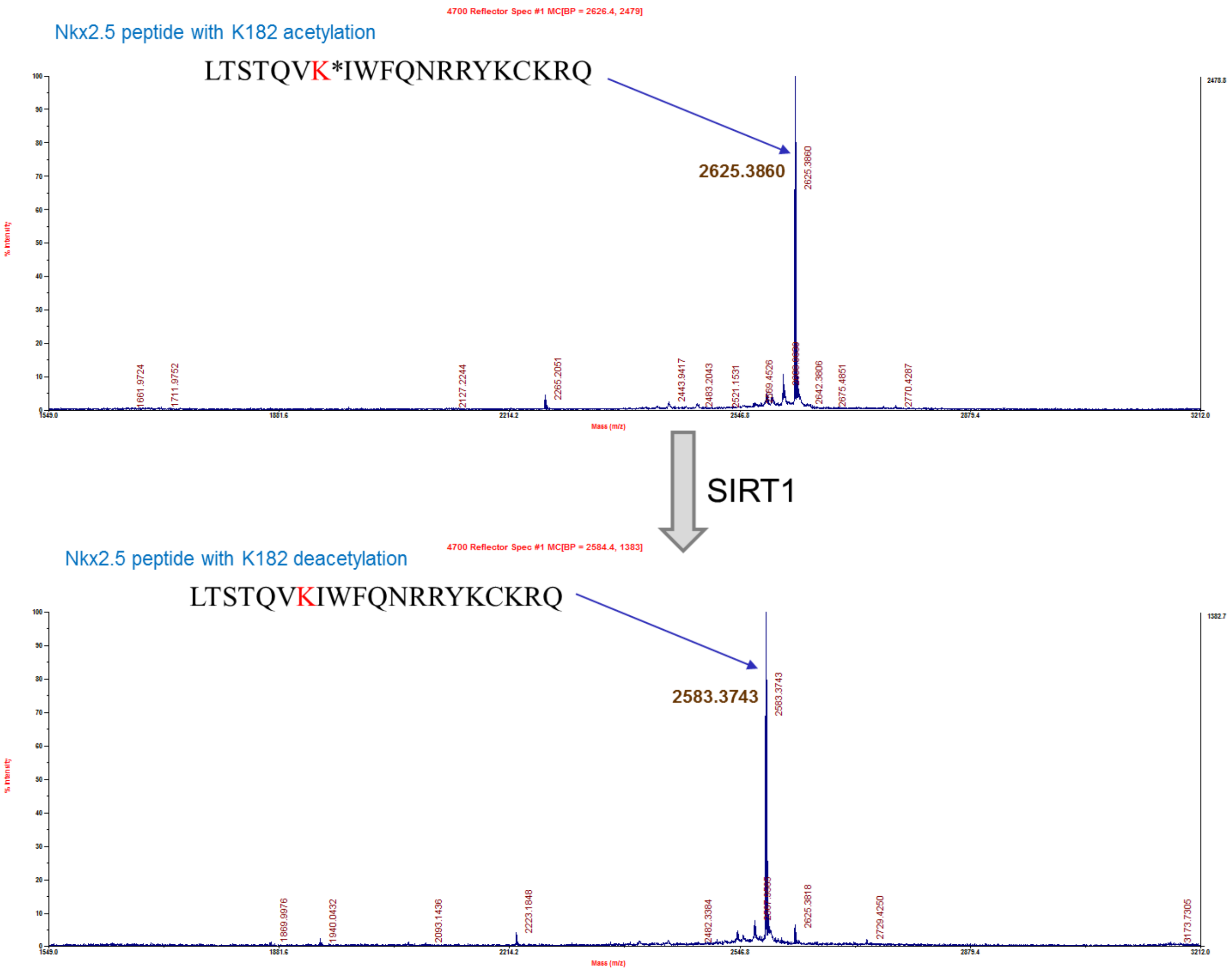

Supplementary Figure 3 Tandem MS analysis of the Nkx2.5 peptides containing acetylated K182 before and after the *in vitro* deacetylation assay.

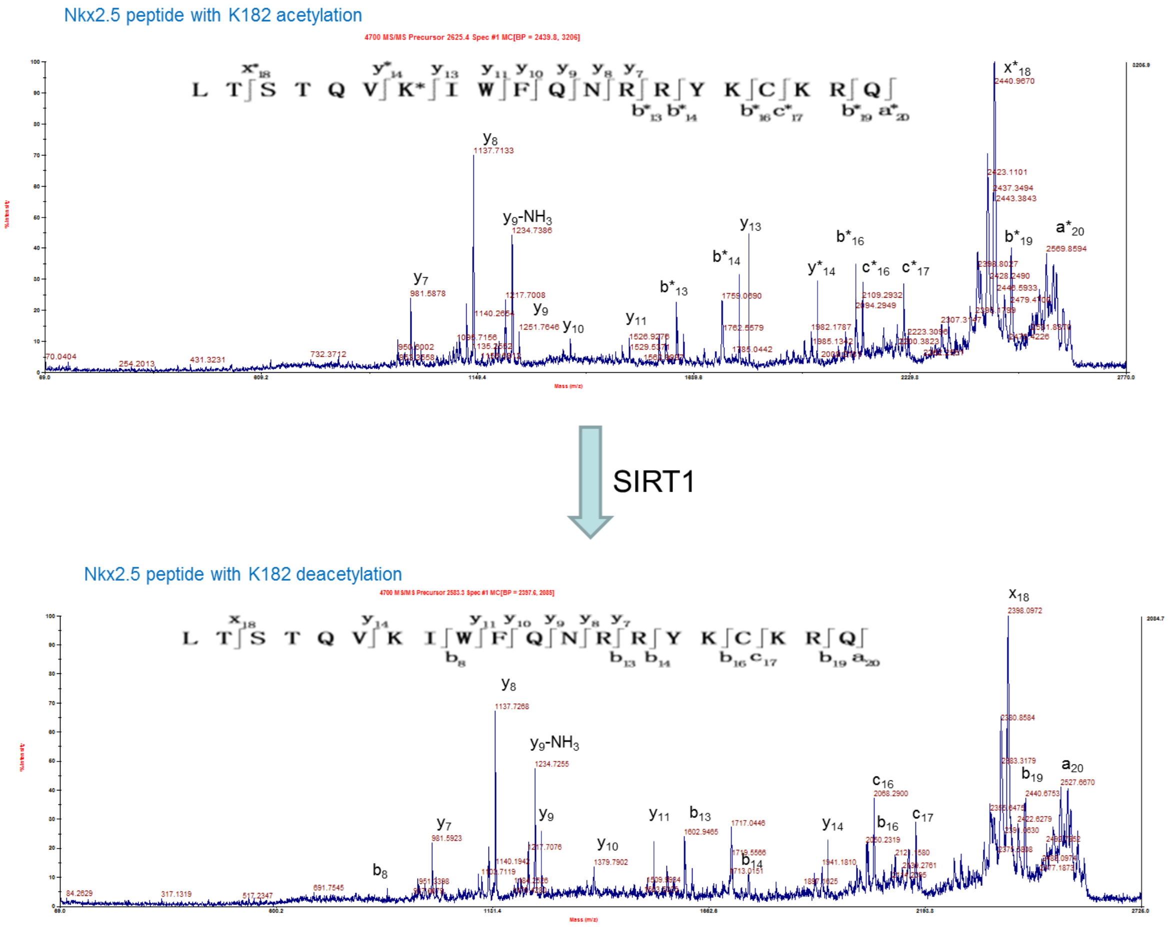

Supplementary Figure 4 Tandem MS (MS/MS) analysis of the Nkx2.5 peptides containing acetylated K182 before and after the *in vitro* deacetylation assay.

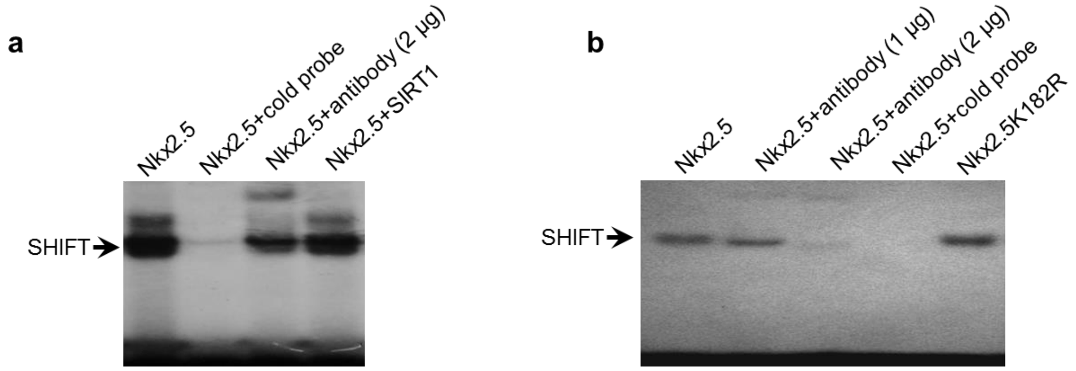

Supplementary Figure 5 SIRT1 or K182R does not affect the DNA-binding ability of Nkx2.5.
(**a**) SIRT1 did not affect the DNA-binding ability of Nkx2.5. HEK293A cells were transfected with Nkx2.5 alone or together with SIRT1. Nuclear proteins were extracted and EMSA was performed with the Nkx2.5 binding element of the *ANF* promoter. Super-shift assay was performed with 2 μg anti-Nkx2.5 antibody.
(**b**) The DNA binding activity of Nkx2.5-K182R mutant is similar to that of WT-Nkx2.5. HEK293A cells were transfected with Nkx2.5, Nkx2.5 mutant (K182R) or control plasmids. Nuclear proteins were extracted, and EMSA was performed with Nkx2.5 binding element of the *ANF* promoter. Super-shift assay was performed with 1 or 2 μg anti-Nkx2.5 antibody.

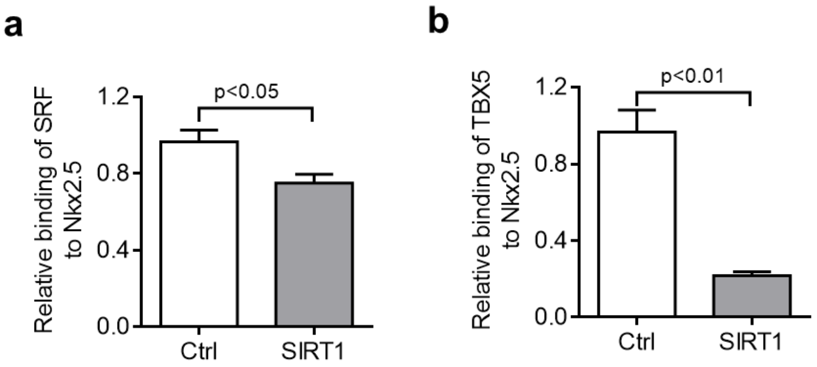

Supplementary Figure 6 SIRT1 inhibits Nkx2.5 binding to its co-factors (related to Figure 7). (**a**) Relative binding of SRF to Nkx2.5 in the presence/absence of SIRT1 (related to Figure 7a). (**b**) Relative binding of TBX5 to Nkx2.5 in the presence/absence of SIRT1 (related to Figure 7b).
